# Antimicrobial use and its association with the presence of methicillin-resistant staphylococci (MRS) and extended-spectrum beta-lactamases (ESBL)-producing coliforms in mastitic milk on dairy farms in the Chiba Prefecture, Japan

**DOI:** 10.1016/j.heliyon.2022.e12381

**Published:** 2022-12-15

**Authors:** Masato Kikuchi, Takuma Okabe, Hideshige Shimizu, Takashi Matsui, Fuko Matsuda, Takeshi Haga, Kyoko Fujimoto, Yuko Endo, Katsuaki Sugiura

**Affiliations:** a Chiba Prefectural Agricultural Mutual Aid Association, Chiba, Japan; b Department of Veterinary Medical Sciences, Graduate School of Agriculture and Life Sciences, The University of Tokyo, Tokyo, Japan; c Laboratory of Environmental Sciences for Sustainable Development, Graduate School of Agriculture and Life Sciences, The University of Tokyo, Tokyo, Japan; d Nippon Institute for Biological Science, Ome, Tokyo, Japan

**Keywords:** Antimicrobial use, Antimicrobial resistance (AMR), Defined daily dose (DDD), Antimicrobial treatment incidence (ATI), Dairy cattle, Japan, Methicillin-resistant staphylococci (MRS), Extended-spectrum beta-lactamase (ESBL)-Producing coliforms, Mastitis

## Abstract

Food-producing animals, including dairy cattle, are potential reservoirs of antimicrobial resistance. However, there is limited data on antimicrobial use and the selection of resistant bacteria. Therefore, we investigated the association between antimicrobial use and resistance to mastitis pathogens using 2016 data from milk samples collected from cows with mastitis in 134 dairy farms in Chiba Prefecture, one of the principal dairy production prefectures in Japan. We recorded the antimicrobial use and isolation of methicillin-resistant staphylococci (MRS) and extended-spectrum beta-lactamase (ESBL)-producing coliforms (*E. coli* and *Klebsiella* spp.), and used the antimicrobial treatment incidence (ATI; the theoretical number of animals per 1000 animal-days subjected to antimicrobial treatment) to indicate antimicrobial use on each farm. The farms in which MRS or ESBL-producing coliforms were isolated from at least one mastitic milk sample were classified as antimicrobial resistance (AMR)-positive, and those in which neither MRS nor ESBL-producing coliforms were isolated were classified as AMR-negative. The AMR-positive farms showed a significantly higher ATI (median 45.17) than AMR-negative farms (median 38.40). The results indicate that high antimicrobial usage is associated with AMR in staphylococci and coliforms isolated from mastitic milk on dairy farms in Chiba Prefecture.

## 1 Introduction

Antimicrobial resistance (AMR) is a global public health concern. Although its development and transmission is complex and not yet fully understood, antimicrobial use is a major driver of AMR (O'Neill, 2016; WHO, 2015). Hence, antimicrobial stewardship is a coordinated program used in human and veterinary medicine. A correlation between antimicrobial use and resistance in food-producing animals, including dairy cattle, has been reported (Oliver, 2011; Chantziaras, 2014). Bacteria are subjected to antimicrobial treatments on dairy farms, and the subsequent selection pressure may result in the selection and dissemination of resistant bacteria (Acar et al., 2006; Silbergeld et al., 2008). The loss of efficacy of antimicrobials due to the presence of resistant bacteria, as seen in human medicine, will also occur in veterinary medicine (European Medicine Agency, 2015). Therefore, it is important to verify if there is association between antimicrobial use and selection of resistant bacteria on dairy farms for both clinical and public health reasons.

Mastitis is one of the primary causes of antimicrobial use in dairy cattle (Mitchell et al., 1998). *S. aureus* and streptococci are frequently isolated from bovine intramammary infections (Olde Riekerink et al., 2008). Antimicrobial therapy is an approach commonly employed to reduce the incidence of mastitis in dairy farms (Erskine et al., 2003), and various antimicrobials have been approved for mastitis therapy in most countries (Ruegg, 2021). Relatively narrow-spectrum antimicrobials that target gram-positive organisms such as *Streptococci* and *Staphylococci*, are the most approved; however, in some countries, broad-spectrum antimicrobials such as 3rd and 4th-generation cephalosporins and some quinolones are approved (Ruegg, 2021). While no 3rd and 4th-generation cephalosporins are approved for mastitis therapy in Japan, β-lactams, aminoglycosides, 14- and 16-membered macrolides, and tetracyclines are approved for intramammary use, and quinolones are approved for parenteral use (Ministry of Agriculture, Forestry and Fisheries, 2014; Shinozuka et al., 2018). However, antimicrobial treatments resulting in failure of bacteriological cure are common in staphylococcal mastitis. AMR is considered as a reason for low cure rates. Resistance to various antimicrobials is commonly seen in bovine staphylococcal mastitis isolates (Barkema et al., 2006).

AMR has also been commonly observed in coliforms isolated from mastitic cows (Lehtolainen et al., 2003). Broad-spectrum antimicrobials, such as quinolones or 3rd or 4th-generation cephalosporins, are commonly used to treat coliform mastitis (including *E. coli* and *Klebsiella* spp). in some countries (Erskine et al., 2002; Suojala et al., 2013; Shinozuka et al., 2018). Resistance to 3rd generation cephalosporins or quinolones in *E. coli* in dairy farms has been reported (Duse et al., 2016; Boireau et al., 2018; Yu et al., 2020). Moreover, the prevalence of extended-spectrum beta-lactamase (ESBL)-producing coliforms in dairy farms has been studied in several countries (Dahmen et al., 2013; Ali et al., 2016; Taniguchi et al., 2021; Weber et al., 2021).

Herd-level association has been reported between antimicrobial use and AMR in bovine mastitis pathogens, such as *Staphylococcus aureus* (Saini et al., 2012), gram-negative bacteria (Saini et al., 2013), and non-aureus *staphylococci* (Nobrega et al., 2018). However, there is little information on the association between antimicrobial use and AMR in mastitis pathogens in Japan. This study evaluated the herd-level association between antimicrobial use and methicillin-resistant staphylococci (MRS) and ESBL-producing coliforms isolated from mastitic milk in dairy farms using data from 134 dairy farms in Chiba Prefecture, Japan.

## 2 Materials and methods

### 2.1 Dairy farms subjected to the analysis in this study

**Figure 1:**
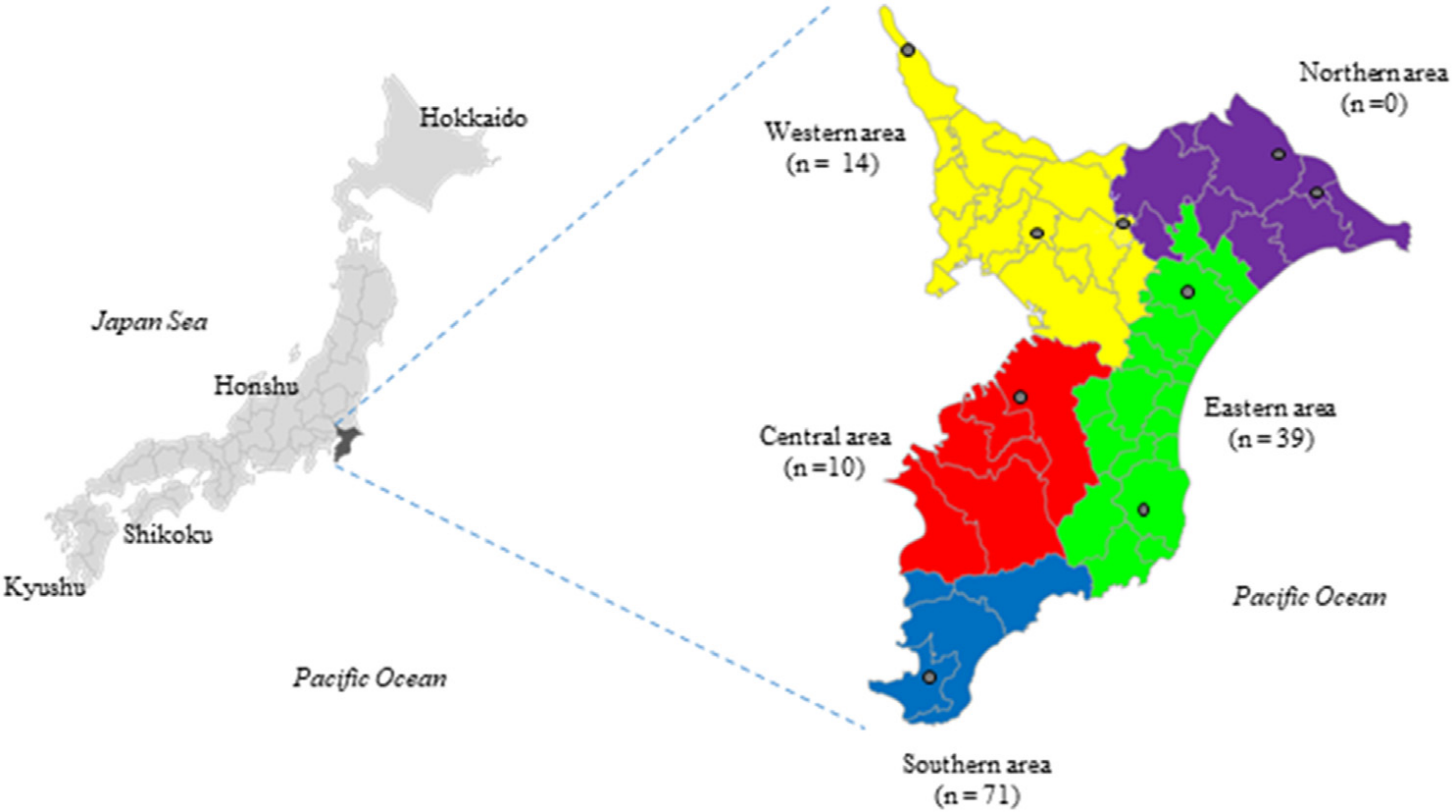
Location of Chiba Prefecture and its five jurisdictional districts Black open circles indicate the location of NOSAI Chiba Veterinary Clinics. Number of farms analyzed in each district is shown in parentheses.

As of February 2016, 720 dairy farms housing 25,100 cows operated in Chiba Prefecture (one of the 47 prefectures in Japan) (Ministry of Agriculture, Forestry and Fisheries, 2017). Chiba Prefectural Agricultural Mutual Aid Association (NOSAI Chiba) has eight veterinary clinics in five districts of Chiba Prefecture (Figure 1). NOSAI Chiba had contracts with 596 of the 720 dairy farms in the prefecture at the end of 2016, providing them with veterinary services, including mastitis treatment. Of these 596 farms, 442 farms maintained data on antimicrobial use and the number of cows in 2016. Of these 442 farms, 134 farms that were subjected to mastitic milk bacterial isolation and drug resistance identification in 2016 were analyzed in this study. NOSAI is a nationwide agricultural insurance scheme that provides contracted dairy and beef cattle, horse, and breeding pig farms with veterinary services and life insurance for dead and culled animals. This study was approved by the Animal Research Ethics Committee of the NOSAI Chiba (approval number CNS140102).

### 2.2 Creation of a database

We constructed a database by entering for each farm data on antimicrobial use, isolation of resistant bacteria and herd size: data on antimicrobial use included the weight of active ingredient (in mg) aggregated from the treatment records for twenty-three microbial agents (grouped into nine classes) and administration routes (injection, intramammary, oral, or intrauterine) (Table 1). The intramammary products for dry-cow therapy (DCT) were entered separately from those with the same constituents for lactating cows. We calculated the data on hard-size (average number of cows kept on the farm) by summing the number of cows over two years of age present on the farm at the beginning of every 21-day period (i.e., the average length of the bovine estrous cycle) of the year and dividing the sum by 18 (the number of cycles in a year).

**Table 1:** Antimicrobial use in 134 farms by antimicrobial agent and administration route (2016).

Antimicrobial class	Antimicrobial agent	Administration route	Number of antimicrobial treatment incidence (ATI) (proportion in percentage)					
			All farms (n = 134)		AMR (+) farms (n = 47)		AMR (-) farms (n = 87)	
Ttracycline	Oxytetracycline	intramammary	0.6	(1.4%)	1	(2.3%)	0.3	(0.9%)
		injection	0.3	(0.7%)	0.4	(0.9%)	0.2	(0.6%)
	Chlortetracycline	intrauterine	0.05	(0.1%)	0.05	(0.1%)	0.05	(0.1%)
Amphenicols	Florfenicol	injection	0.02	(0.04%)	0.02	(0.03%)	0.02	(0.04%)
Penicillins	Amoxicillin	injection	0.0005	(0.001%)	0.0001	(0.0002%)	0.0007	(0.002%)
	Ampicillin	injection	3.2	(7.7%)	3.7	(8.1%)	3	(7.5%)
		intrauterine	0.2	(0.5%)	0.2	(0.5%)	0.2	(0.5%)
		peroral	0.1	(0.3%)	0.1	(0.3%)	0.1	(0.3%)
	Dicloxacillin	intramammary	0.2	(0.4%)	0.2	(0.4%)	0.2	(0.4%)
	Mecillinam	injection	0.07	(0.2%)	0.06	(0.1%)	0.08	(0.2%)
	Penicillin	intramammary	12	(27.6%)	12	(26.5%)	11	(28.3%)
		injection	0.7	(1.8%)	0.7	(1.5%)	0.8	(1.9%)
Sulfonamides	Sulfadimethoxine	injection	0.03	(0.08%)	0.05	(0.1%)	0.02	(0.06%)
	Sulfamonomethoxine	peroral	0.006	(0.01%)	0.01	(0.03%)	0.002	(0.006%)
Macrolide	Erythromycin	intramammary	0.02	(0.06%)	0.03	(0.07%)	0.02	(0.05%)
	Tilmicosin	peroral	0.1	(0.3%)	0.1	(0.2%)	0.1	(0.3%)
		injection	0.007	(0.02%)	0.007	(0.01%)	0.007	(0.02%)
	Tylosin	injection	0.2	(0.5%)	0.3	(0.6%)	0.1	(0.4%)
Aminoglycoside	Dihydrostreptomycin	intramammary	11	(27.1%)	12	(25.9%)	11	(27.8%)
		injection	0.1	(0.3%)	0.1	(0.3%)	0.1	(0.3%)
	Kanamycin	intramammary	0.2	(0.4%)	0.2	(0.5%)	0.1	(0.3%)
		injection	0.02	(0.05%)	0.03	(0.06%)	0.02	(0.04%)
Cephalosporin	Cefapirin	intramammary	0.005	(0.01%)	0.01	(0.03%)	0	(0.0%)
	Cefazolin	intramammary	10	(24%)	11	(23.8%)	9.6	(24.2%)
	Cefazolin	injection	1.4	(3.2%)	1.6	(3.4%)	1.2	(3.1%)
	Cefuroxime	intramammary	1.2	(2.8%)	1.7	(3.8%)	0.9	(2.2%)
Trimetoprim	Ormetoprim	peroral	0.006	(0.02%)	0.01	(0.03%)	0.003	(0.007%)
Quinolones	Danofloxacin	injection	0.001	(0.003%)	0.004	(0.008%)	0	(0.0%)
	Enrofloxacin	injection	0.1	(0.3%)	0.2	(0.4%)	0.1	(0.3%)
	Orbifloxacin	injection	0.07	(0.2%)	0.08	(0.2%)	0.07	(0.2%)
Used for DCT			18.44	(43.8%)	19.41	(42.1%)	17.92	(44.9%)
Total			42.06	(100.0%)	46.06	(100.0%)	39.90	(100.0%)

### 2.3 Quantification of antimicrobial use on dairy farms

Antimicrobial treatment incidence (ATI) was used to measure the antimicrobial use in each dairy farm. The ATI presents the theoretical number of animals per 1000 animal-days subjected to antimicrobial treatment, assuming that the antimicrobial products are used in a cow of standard weight according to the dosage specified in the SPC (summary of the product characteristics). We first calculated the number of defined daily doses (DDDs) of the antimicrobial agent *a* (weight of biomass subjected to treatment with an antimicrobial agent *a* in kg-days) by dividing the weight of the active ingredient by the DDD of the antimicrobial agent. The DDDs used are available from the Japanese DDD values of antimicrobial agents (DDDjp) (Fujimoto et al., 2021). The ATI for antimicrobial agent α (ATIα) was calculated by dividing the number of DDDs by the average number of cows on the farm and the standard weight of dairy cows (635 kg), as in Eq. (1).

(1) $${ATI}_{a}=\frac{{Number\;of\;DDDs\;for\;antimicrobial}_{a}\left(kg・days\right)}{Average\;number\;of\;cows\;on\;the\;farm\times 635\left(kg\right)\times 365\left(days\right)}\times 1000\left(animals\right)$$

The overall antimicrobial use on each farm was then calculated by summing the ATI for the antimicrobial agents used on that farm, as in Eq. (2).

(2) $$ATI=\sum_{a}{ATI}_{a}$$

### 2.4 Isolation of MRS and ESBL-producing coliforms

Mastitis cows were detected by farmers during milking by visual observation of abnormal milk (flakes, fibrin clots, or abnormal milk color) and/or inflammatory changes in the udder (swelling, hardness, heat, pain, or redness). Before treatment, milk samples were collected in sterilized tubes from the affected quarter(s) by trained farmers or veterinarians. The samples were stored at 4 °C or frozen at −20 °C and immediately subjected to a bacterial isolation test. Bacteriological examinations were outsourced to the Sanritsu Zelkova Veterinary Laboratory (Kanagawa, Japan). After one predominant bacterium was isolated from the milk sample, identification, and antimicrobial susceptibility testing of staphylococci and coliforms (*E. coli* and *Klebsiella. spp*) were conducted using a MicroScan WalkAway Plus System (Beckman Coulter, USA), an automated identification system for most gram-positive cocci and gram-negative rods. The broth microdilution method was applied for susceptibility testing according to the Clinical and Laboratory Standards Institute (CLSI) guidelines (CLSI, 2012). The identification of multidrug-resistant bacteria was based on the CLSI guidelines and criteria. *Staphylococcus* isolates were identified as MRS or MRSA (methicillin-resistant *Staphylococcus aureus*) using the detection of oxacillin and cefoxitin resistance with minimum inhibitory concentration (MIC) interpretive criteria ≥4 μg/mL for oxacillin and ≥8 μg/mL for cefoxitin in *S. aureus* and *S. lugdunensis,* and ≥0.5 μg/mL for oxacillin in coagulase-negative staphylococci (CNS), except *S. lugdunensis*. For cases that were difficult to detect using oxacillin resistance only (MICs 0.5–2.0 μg/mL), mecA gene detection was conducted using PCR assay. Based on the National Institute of Infectious Diseases guidelines (NIID, 2019), mA1 (5'-TGCTATCCACCCTCAAACAGG-3’) and mA2 (5’AACGTTGTAACCACCCCAAGA-3’) primers were used for mecA PCR with initial denaturation conditions of 94 °C for 1 min, followed by 30 cycles of denaturation at 94 °C for 1 min, annealing at 50 °C for 30 s, and elongation at 72 °C for 2 min. Coliform (*E. coli* and *Klebsiella* spp*.*) isolates were identified as ESBL-producing using the broth microdilution method, followed by testing the hydrolysis of chromogenic oxyimino-cephalosporin HMRZ-86 using the Cica Beta Test (Kanto Chemical, Tokyo, Japan).

### 2.5 Statistical analysis

Farms in which either MRS or ESBL-producing coliforms were isolated from at least one mastitic milk sample were classified as AMR-positive, and those with neither MRS nor ESBL-producing coliforms were classified as AMR-negative. The Wilcoxon rank-sum test was used to compare the average herd size and ATI between AMR-positive and AMR-negative farms. Statistical analysis was conducted using R Statistical Software version 3.6.1 (R Foundation for Statistical Computing, Vienna, Austria).

## 3 Results and discussion

### 3.1 Antimicrobial use

The average antimicrobial use on 134 dairy farms, measured using ATI, was 42.06 (Table 1), of which, approximately 84% of antimicrobials were intramammary-administered to dry cows (44%) and lactating cows (40%). This was followed by injection (14%) and oral administration (2%). The most frequently used antimicrobial was penicillin for intramammary use with the ATI of 12, followed by dihydrostreptomycin for intramammary use (ATI of 11), cefazolin for intramammary use (ATI of 10), ampicillin for injection (ATI of 3.2), cefazolin for injection (ATI of 1.4), cefuroxime for intramammary use (ATI of 1.2), penicillin for injection (ATI of 0.7), oxytetracycline for intramammary use (ATI of 0.6) and oxytetracycline for injection (ATI of 0.3), ampicillin for intrauterine use (ATI of 0.2).

Antimicrobial use on dairy farms has been previously assessed using ATI in the USA and Belgium (de Campos et al., 2021; Stevens et al., 2016). Our results revealed that the dairy farms in Chiba Prefecture analyzed in this study used twice as much antimicrobials as those in Wisconsin, USA and Flanders, Belgium, where the overall ATI antimicrobial use was 17.2 in 2017 and 20.8 in 2012–2013, respectively (de Campos et al., 2021; Stevens et al., 2016).

### 3.2 Antimicrobial resistance

To our knowledge, this study is the first to reveal an association between antimicrobial use assessed using ATI and AMR in pathogens isolated from mastitic milk in Japan. Of the 134 farms studied, MRS was isolated from 40, and ESBL-producing coliforms were isolated from 7 farms. Neither MRS nor ESBL-producing coliforms were isolated from 87 of the 134 farms. In terms of herd size (the number of cows on the farm), there was no significant difference between AMR-positive and AMR-negative farms, with the mean number of cows 38.2 and 36.2, respectively.

Of the 210 CNS isolates, 83 (39.5%) were methicillin-resistant (Table 2). The proportion of MRS in bovine mastitic milk was higher than previously reported in other countries (Fessler et al., 2010; Gindonis et al., 2013; Kim et al., 2019; Sampimon et al., 2011; Sawamt et al., 2009). Although MRS is a suspected reservoir of the mecA gene in staphylococci (Hanssen et al., 2007), whether there was a clonal expansion of MRS is unclear because the genetic diversity of the mecA gene was not analyzed in this study. Further studies involving genetic analysis of MRS are needed to reveal the distribution of methicillin-resistant genes in milk from cows with mastitis in dairy farms in Chiba Prefecture.

**Table 2:** Number of mastitic milk samples from which Staphylococci, *Escherichia coli,* or *Klebsiella* spp. were isolated from cows with clinical mastitis (n = 1010) in Chiba Prefecture, Japan, dairy farms.

Bacterial species	Number of isolates	Number of multidrug-resistant isolates
Coagulase-negative staphylococci (CNS)	210	83∗
*Staphylococcus aureus*	110	0∗
*Escherichia coli* (*E. coli*)	65	6∗∗
*Klebsiella* spp.	13	2∗∗

∗ Number of methicillin-resistant isolates.

∗∗ Number of extended-spectrum beta-lactamase (ESBL)-producing isolates.

The proportions of ESBL-producing coliforms of the coliform isolates (6 of 65 *E coli* and 2 of 15 *Klebsiella* spp) (Table 2). were considerably higher than previously reported in Japan and elsewhere. Ohnishi et al. (2013) reported that 65 CTX-M-2/15/14 ESBL-producing Enterobacteriaceae were isolated from 258,888 mastitic milk samples from Japanese dairy farms between 2007 and 2011. This proportion was lower (0.4%) in France (Dahmen et al., 2013). Locatelli et al. (2010) reported that only one out of 140 *Klebsiella* isolates from milk samples between 2008 and 2009 contained CTX-M1 ESBL-producing bacteria. Freitag et al. (2017) reported that 12/878 (1.4%) of unrelated *E. coli* from mastitis cases in Germany were ESBL-producing, implying that the frequency of ESBL-producing coliforms is higher in dairy farms in Chiba Prefecture than in other prefectures in Japan and other countries.

### 3.3 Association between antimicrobial use and resistance

Table 3 shows the ATIs of AMR-positive and AMR-negative farms and the Wilcoxon rank-sum test results. The overall ATI of AMR-positive farms was significantly higher than that of AMR-negative farms, with median ATI values of 45.17 and 38.40, respectively (*P* = 0.023). The ATIs of antimicrobials intramammary-administered for lactating cows and those administered by injection in AMR-positive farms were also significantly higher than those in AMR-negative farms, with median values of 15.29 vs. 11.37 (*P* = 0.045) and 6.67 vs. 4.79 (*P* = 0.021) respectively. This suggests that under field conditions the antimicrobial use is positively associated with AMR in bovine mastitis pathogens.

**Table 3:** Antimicrobial treatment incidence (ATI) medians in antimicrobial resistance (AMR)-positive and AMR-negative farms.

		Number of antimicrobial treatment incidence (ATI)		
		AMR-positive farms (n = 47)	AMR-negative farms (n = 87)	*p*-value
Administration route	Intramammary (for dry cow therapy)	20.09	19.17	0.22
	Intramammary (for lactating cow)	15.29^a^	11.37^b^	0.045
	Injection	6.67^a^	4.79^b^	0.021
	Oral	0.02	0.00	0.23
	Intrauterine	0.06	0.00	0.29
Antimicrobial class	Tetracyclines	0.93^a^	0.34^b^	0.00016
	Amphenicol	0	0	0.27
	Penicillins	17.30^a^	14.62^b^	0.04
	Sulfonamides	0.01^a^	0^b^	0.018
	Macrolides	0.04	0	0.071
	Aminoglycosides	12.40	11.29	0.19
	Cephalosporins	12.51	9.74	0.051
	Trimetoprim	0	0	0.33
	Quinolones	0.12	0.05	0.065
Total		45.17^a^	38.4^b^	0.023

AMR-positive farms: farms in which methicillin-resistant staphylococci (MRS) or extended-spectrum β-lactamase (ESBL)-producing coliforms (*E. coli* and *Klebsiella* spp.) were detected in at least one mastitic milk sample.

AMR-negative farms: farms in which neither MRS nor ESBL-producing coliforms were detected in the mastitic milk samples.

a - b: significant difference between AMR-positive and AMR-negative farms (p < 0.05, Wilcoxon rank-sum test).

There is an ongoing debate on whether antimicrobial use is a risk factor in selecting resistance to *S. aureus* in dairy farms (Schnitt and Tenhagen, 2020). A Canadian study revealed a positive correlation between intramammary and systemically administered penicillin treatments and emergence of resistant *S. aureus* in dairy farms (Saini et al., 2012). In contrast, Oliver et al. (2011) concluded no association between antimicrobial use in adult dairy cows and resistant veterinary and human pathogens. studies in Germany and the UK have suggested an association between the use of 3rd or 4th generation cephalosporins and the presence of ESBL-producing *E. coli* (Gonggrijp et al., 2016; Snow et al., 2012), whereas a study in the Netherlands concluded no association between antimicrobial use and ESBL-producing *E. coli* (Santman-Berends et al., 2017).

Our results did not reveal a significant correlation between the presence of resistant bacteria (MRS or ESBL-producing coliforms) and the use of intramammary antimicrobial used for DCT, whereas a previous Canadian study reported that the herd-level use of intramammary-administered penicillin for DCT (penicillin-novobiocin combination) was positively associated with AMR in *S. aureus* isolated from bovine mastitic milk using a multivariable logistic regression model (Saini et al., 2012), possibly because most dairy farms in Chiba Prefecture practice blanket dry-cow therapy (BDCT) for most cows regardless of their antimicrobial resistance status. Forty-five of 47 AMR-positive farms and 82 of 87 AMR-negative farms practiced BDCT.

Use of antimicrobials administrated intrammamarily for lactating cows reflects the frequency of treatment for clinical mastitis. Our results indicated a higher incidence of antimicrobial treatment for clinical mastitis in AMR-positive farms than in AMR-negative. This was consistent with the results of previous studies conducted in the USA and Canada (Nobrega et al., 2018; Sani et al., 2012). Nobrega et al. (2018) reported that AMR in non-*Aureus* staphylococci isolated from milk was associated with systemic but not intramammary administration of antimicrobials. Saini et al. (2012) reported a positive correlation between intramammary and systemically administered penicillin and AMR in mastitis *S. aureus* isolates.

Our results also revealed that injection-administered antimicrobials could affect AMR in udder pathogens (Table 3). Administered antimicrobials must reach the site of infection in an effective concentration in order form them to be effective. However, attaining and maintaining therapeutic concentrations of most antimicrobials in udder tissue or milk following systemic administration is difficult (Pyörälä, 2009). Furthermore, although the bovine udders have a rich blood supply, the rate of translocation of antimicrobials into milk following parenteral administration is affected by the lipid solubility and plasma-protein binding (Baggot, 2006). It is only the lipid-soluble, non-ionized, and plasma protein-unbound fraction of antimicrobials that can cross the blood-milk barrier to enter milk and disperse into the transcellular fluid. Penicillins do not easily cross biological membranes, as they are predominantly ionized in the plasma and less lipid-soluble. Thus, the therapeutic concentration of penicillin achieved in the udder following systemic administration might lead to the selection of staphylococci that are penicillin- and ampicillin-resistant.

Among the antimicrobial classes, the penicillins, tetracyclines, and sulfonamides were used more frequently in AMR-positive than in AMR-negative farms, with median ATIs of 17.30 vs. 14.62 (*P* = 0.04), 0.93 vs. 0.34 (*P* = 0.00016), and 0.01 vs. 0.00 (*P* = 0.018), respectively.

Penicillins include penicillin, the most used antimicrobials administrated intrammamarily and ampicillin, the most used antimicrobials administrated by injection and intrauterine route. Overuse of penicillins might increase the selective pressure for resistant bacteria, possibly resulting in the occurrence of MRS.

**Figure 2:**
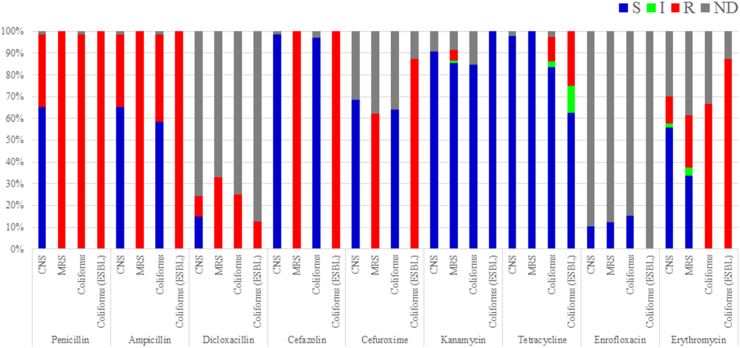
Distribution of antimicrobial sensitivity test results for coagulase-negative staphylococci (CNS), methicillin-resistant CNS (MRS), coliforms and extended-spectrum beta-lactamase (ESBL)-producing coliforms (coliforms (ESBL)) isolated from mastitic milk samples. Broth microdilution method was used to test bacteria for antimicrobial sensitivity. Sensitivity test results categorized as sensitive (blue), intermediate (green), resistant (red), and no data (gray).

The relationship between tetracycline overuse and emergence of MRSA in pig farms has been reported by Larsen et al. (2016) and van Duijkeren et al. (2008). That is considered to be caused by co-selection by which bacteria acquire tetracycline resistant gene such as *tet*(K) and methicillin resistant genes simultaneously. However, this might be not the case with the results in this study because the sensitivity tests on bacteria isolated from mastitic milk in this study revealed that none of the 83 MRS and only 2 of the 8 ESBL-producing coliforms (*Escherichia coli* or *Klebsiella* spp.) exhibited tetracycline resistance (Figure 2). However, the actual prevalence of AMR in the farms was unclear in this study because the data available was limited to a part of bacteria isolated from mastitic milk. Further studies including genetic analyses are needed.

A limitation of this study is that we only investigated the association between antimicrobial use and MRS or ESBL-producing coliforms. Potential risk factors other than antimicrobial use that might affect the emergence and selection of AMR were not considered because data were not available. Although the herd size of each farm was a risk factor in this study (Schnitt and Tenhagen, 2020), it was not significantly different between AMR-positive and AMR-negative farms. Other potential risk factors include average herd parity, barn type, age, and body weight (Rajala-Schultz et al., 2004; Sol et al., 2000). Further studies are required to accurately determine the effects of antimicrobial use on AMR.

## Declarations

### Author contribution statement

Masato Kikuchi: Conceived and designed the experiments; Performed the experiments; Analyzed and interpreted the data; Wrote the paper.

Takuma Okabe, Hideshige Shimizu and Takashi Matsui: Performed the experiments.

Kyoko Fujimoto and Yuko Endo: Analyzed and interpreted the data.

Fuko Matsuda, Takeshi Haga and Katsuaki Sugiura: Conceived and designed the experiments; Wrote the paper.

### Funding statement

Prof Katsuaki Sugiura was supported by 10.13039/100015103Japan Racing Association [Project No. 3-Keichikushin-109].

### Data availability statement

Data will be made available on request.

### Declaration of interest’s statement

The authors declare no conflict of interest.

### Additional information

No additional information is available for this paper.
